# Parliament and the Profession

**Published:** 1916-01-08

**Authors:** 


### PARLIAMENT AND THE PROFESSION.

### Safe Conducts for German Doctors.

THE number of enemy doctors and Red Cross workers who have been allowed to go through our naval blockade under safe conducts to Europe is eighty-six, and the number of those who have been allowed to pass without safe conduct is sixty-nine. Lord Robert Cecil, who made a statement to this effect, added that the Government had not so far felt obliged to refuse requests made to them on the ground of the Geneva Convention for the free passage of enemy doctors and Red Cross employees returning to their own country from overseas. In most cases Red Cross workers captured or detained by the Germans had been also released, but as in some cases they had not been released the Government was considering the advisability of detaining similar German individuals who would otherwise be returned.

### Red Cross Men of Military Age.

MANY Red Cross men of military age are, according to Mr. Evelyn Cecil, being retained in the London Ambulance Column, with the promise that certificates of indispensability will be granted them, which there is every reason to believe will be effective with the recruiting tribunals, notwithstanding that many men over military age are being thrown out of the London Ambulance Column. Mr. Tennant, whose attention has been drawn to the matter, states that there is no reason to think that such certificates of indispensability would have any influence with local tribunals in view of the fact that the Red Cross Society has arranged with Lord Derby to release every available man possible for military service.

### The Treatment of Defective Recruits.

MR. BOYTON asked the Under-Secretary for War if he was aware that a number of recruits were rejected for minor physical defects, many of which could be successfully dealt with in hospitals; and if the Government would consider the desirability of paying those who offered to undergo operations or treatment at Army rates while they are detained in hospital, provided that they have previously attested. Mr. Forster replied that the number of men rejected for physical defects of a kind which could be cured by surgical operations was inconsiderable, and the risk and disadvantages of the course suggested were so serious that he feared he could not adopt it. It is difficult to reconcile this statement with the fact that at Guy's Hospital something like 1,000 men who were previously ineligible for the Army have been treated and rendered fit for service, and that similar treatment has been given at many other hospitals.

### Other Matters of Interest.

GRANTS TO OFFICERS' HOSPITALS.—Replying to a question with regard to grants to officers' hospitals, Mr. Forster stated that where assistance from public funds is asked for, each case is considered on its merits.

SANATORIUM BENEFIT.—Mr. Roberts stated that the amount available for the defraying of sanatorium benefit under the National Insurance Act for the period July 15, 1912, to December 31, 1914, was £2,125,000, in addition to which various special grants had been made.

FEMALE NURSES IN MENTAL HOSPITALS.—Replying to Mr. Jowett, Sir John Simon stated that the employment of female nurses in mental hospitals to release men for enlistment was allowed in suitable cases.

DENTISTS WITH THE ARMY.—There are, according to Mr. Tennant, forty-three dentists employed with the Expeditionary Force in France.

